# Impact of exercise on sexual health, body image, and therapy‐related symptoms in women with metastatic breast cancer: The randomized controlled PREFERABLE‐EFFECT trial

**DOI:** 10.1002/ijc.35429

**Published:** 2025-04-03

**Authors:** Martina E. Schmidt, Anouk E. Hiensch, Johanna Depenbusch, Evelyn M. Monninkhof, Jon Belloso, Dorothea Clauss, Nadira Gunasekara, Mark Trevaskis, Helene Rundqvist, Joachim Wiskemann, Jana Müller, Maike G. Sweegers, Andreas Schneweiss, Renske Altena, Joanna Kufel‐Grabwska, Rhodé M. Bijlsma, Lobke van Leeuwen‐Snoeks, Daan ten Bokkel Huinink, Gabe Sonke, Susanne Brandner, Peter Savas, Yoland Antill, Michelle White, Nerea Ancizar, Elsken van der Wall, Neil K. Aaronson, Elzbieta Senkus, Ander Urruticoechea, Eva M. Zopf, Wilhelm Bloch, Martijn M. Stuiver, Yvonne Wengström, Anne M. May, Karen Steindorf

**Affiliations:** ^1^ German Cancer Research Center (DKFZ) and National Center for Tumor Diseases (NCT) Heidelberg, A Partnership Between DKFZ and University Medical Center Heidelberg Heidelberg Germany; ^2^ University Medical Center Utrecht Utrecht University Utrecht The Netherlands; ^3^ Gipuzkoa Cancer Unit, OSID‐Onkologikoa, BioGipuzkoa, Osakidetza San Sebastian Spain; ^4^ German Sport University Cologne Cologne Germany; ^5^ Mary MacKillop Institute for Health Research Australian Catholic University Melbourne Australia; ^6^ Karolinska Institutet and Karolinska Comprehensive Cancer Center Karolinska University Hospital Stockholm Sweden; ^7^ Heidelberg University Hospital and NCT Heidelberg Heidelberg Germany; ^8^ Netherlands Cancer Institute Amsterdam The Netherlands; ^9^ Greater Poland Cancer Centre Poznan Poland; ^10^ Diakonessenhuis Utrecht The Netherlands; ^11^ Alexander Monro Ziekenhuis Bilthoven The Netherlands; ^12^ St. Elisabeth‐Krankenhaus GmbH Cologne Germany; ^13^ Royal Melbourne Hospital Melbourne Australia; ^14^ Peter MacCallum Cancer Centre and Sir Peter MacCallum Department of Oncology The University of Melbourne Melbourne Australia; ^15^ Cabrini Cancer Institute Cabrini Health Malvern Australia; ^16^ Medical University of Gdańsk Gdańsk Poland

**Keywords:** exercise, metastatic breast cancer, quality of life, sexual health, supportive care

## Abstract

The understanding and treatment of sexual health problems, impaired body image, and other non‐life threatening but burdensome symptoms of women with metastatic breast cancer (mBC) is still insufficient. We studied the factors associated with such symptoms and investigated whether these problems could be alleviated by a structured exercise intervention. In the multinational PREFERABLE‐EFFECT study, 355 women with mBC were randomly assigned to usual care (*n* = 178) or a 9‐month supervised exercise program (*n* = 177). Breast cancer‐specific functions and symptoms (EORTC QLQ‐BR42) were assessed at baseline, 3, 6 (primary timepoint), and 9 months. Linear regression models and linear mixed models for repeated measures were calculated. At baseline, participants were 55.4 ± 11.2 years old, 52.4% were undergoing endocrine therapy, and 25.4% chemotherapy. Baseline sexual functioning was low, with 94.3% reporting no or little sexual activity. Age and depressive symptoms were negatively associated with sexual functioning. Among sexually active women, 46.2% felt no or little sexual enjoyment and 37.3% suffered from vaginal dryness. Body image was reported as low by 23.7%. Exercise significantly improved sexual functioning (6‐months between‐group difference (BGD) = 5.6, 95% CI [1.9, 9.4], effect size (ES) = 0.28) and vaginal symptoms (BGD = −7.1 [−11.7, −2.5], ES = 0.25), compared to usual care. Effects on body image were marginal (BGD = 4.0 [−0.2, 8.3], ES = 0.14). Among participants undergoing chemotherapy (*n* = 90), exercise reduced chemotherapy side‐effects (BGD = −8.2 [−15.4, −1.0], ES = 0.48). In conclusion, women with mBC often experience sexual and vaginal problems and other treatment‐related side‐effects. A 9‐month supervised exercise program vs. control was effective in improving sexual functioning and vaginal symptoms among women with mBC.

## Graphical abstract

What's new?

Women with metastatic breast cancer often experience non‐life‐threatening but unpleasant symptoms, including low sexual enjoyment and vaginal dryness, but these symptoms have not been well‐studied and treatments are lacking. Here, the authors conducted a large, multinational randomized controlled trial of exercise in women with metastatic breast cancer. After 9 months, women participating in the supervised, structured exercise program found that their symptoms improved, including a reduction of chemotherapy side effects.

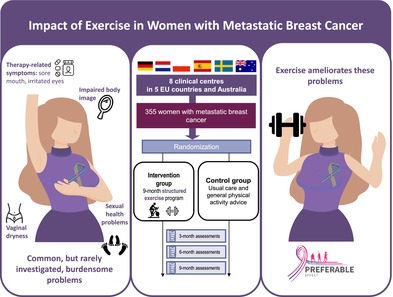

**Abbreviations**
ACSM: American College of Sports Medicine
BC: breast cancer
BGD: between‐group difference
BMI: body mass index
CBT: cognitive behavioral therapy
CG: control group
EORTC: European Organisation for Research and Treatment of Cancer
ES: effect size
ESMO: European Society for Medical Oncology
IG: intervention group
mBC: metastatic breast cancer
RCT: randomized controlled trial
SD: standard deviation

## 1 INTRODUCTION

Metastatic breast cancer (mBC) necessitates complex and prolonged treatment with debilitating side effects affecting patients' quality of life.^1^ In contrast to side effects for which treatment recommendations are well described and widely recognized (e.g., neutropenia, nausea, or vomiting), other burdensome side effects have received less consideration, including sexual health problems and impaired body image.^2^ Chemotherapy, endocrine therapy, and radiation therapy can each lead to sexual dysfunction, such as reduced libido and enjoyment, vaginal dryness, and pain during intercourse (dyspareunia).^3^, ^4^ Anxiety and depression can further exacerbate sexual problems and impair intimate relationships.^3^ Breast surgery, typically conducted during early stage disease, may still impact the sensitivity in the breast area and breast appearance. Endocrine therapy and therapy‐induced menopause can cause, among others, hot flushes, mood swings, weight gain, and joint pain.^3^, ^5^ Currently, the awareness and understanding of sexual health problems, body image issues, and other cancer therapy‐related symptoms in women with advanced breast cancer are still limited.

Exercise has been widely recognized for its multifaceted benefits, which include improving physical fitness, alleviating fatigue, and enhancing psychological well‐being.^6^ While the majority of exercise trials have been conducted in the non‐metastatic disease setting, recently the PREFERABLE‐EFFECT trial showed significant effects of exercise on fatigue, pain, dyspnea, and quality of life for patients with mBC.^7^ We hypothesized that exercise may have further beneficial effects, possibly improving body image and mitigating sexual health problems and other symptoms such as skeletal symptoms, hot flushes, or weight gain that often accompany mBC treatment.^8^, ^9^, ^10^ However, the evidence of the effects of exercise on these potential problems in patients with mBC is still inconclusive, especially regarding sexual health and its underlying or influencing factors.^8^, ^9^, ^11^

Thus, the aim of this study was to investigate the factors associated with problems such as impaired sexual health, body image, and cancer therapy‐related side effects in women with mBC, and to investigate whether these problems could be ameliorated by exercise.

## 2 METHODS

### 2.1 Study design and participants

From 01/2020–08/2022, the multinational randomized controlled PREFERABLE‐EFFECT trial recruited patients with mBC at eight study centers in Germany, the Netherlands, Poland, Spain, Sweden, and Australia, who were eligible if: ≥18 years of age; ECOG performance status ≤2; able and willing to participate in the exercise program. Exclusion criteria were: unstable bone metastases; untreated symptomatic brain metastases; estimated life expectancy <6 months; serious active infection; being too physically active (>210 min/week of moderate‐to‐vigorous exercise) or already engaging in an exercise program comparable to the study intervention; any contraindication for exercise; any circumstances that would impede adherence to study requirements or the ability to give informed consent; or pregnancy. Details of the study design, the full protocol as well as the primary results have been published previously.^7^, ^12^

Participants were randomized (1:1) to either a 9‐month exercise intervention group (IG) or a control group (CG), using a computerized blocked randomization procedure stratified by study center and therapy line (1st/2nd vs. 3rd or higher). Due to the nature of exercise interventions, blinding of participants, local clinicians, or coordinating study team members was not possible. However, baseline assessments were unaffected by group assignment, as group allocation was only revealed upon completion of baseline assessments.

### 2.2 Intervention and control group

The 9‐month exercise intervention (https://www.h2020preferable.eu/exercise-program/) included two 1‐hour exercise sessions per week that were supervised by qualified exercise professionals and comprised resistance, aerobic, and balance exercises. After 6 months, one supervised session was replaced by one unsupervised session. The aim was to gradually increase the exercise intensity during the program, but the intensity was continuously adjusted based on the health status and perceived exertion of the participant. Additionally, participants were encouraged to be physically active for at least 30 min per day on all remaining days of the week. To support this, participants received an activity tracker (i.e., Fitbit Inspire HR) and an exercise app.

The CG received usual care, supplemented with written information on current physical activity guidelines for people diagnosed with cancer (i.e., 150 minutes of aerobic exercise and 2–3 resistance training sessions per week). They were also given a Fitbit activity tracker and were advised to be as physically active as their health allowed.

### 2.3 Assessments

Here we report on secondary outcomes of the PREFERABLE‐EFFECT study. At baseline and 3, 6, and 9 months post‐baseline, participants completed the European Organisation for Research and Treatment of Cancer (EORTC) Quality of Life Breast Cancer Module (QLQ‐BR45), which is an extension of the BR23. In parallel to our trial, the BR45 was further developed by the EORTC resulting in removal of the 3 items “vaginal dryness”, “weight gain perceived as problem”, and “pain in muscles” (i.e., now called BR42) and in some changes in scoring.^13^ For our analyses, we used the new BR42 scoring including 5 functional scores (i.e., sexual functioning, sexual enjoyment (if sexually active), breast satisfaction, body image, and future perspective) and 8 symptom scores (i.e., vaginal symptoms, weight gain, skeletal symptoms, hand/foot/neuropathy, breast symptoms, arm symptoms, chemotherapy side‐effects, and endocrine symptoms). For the vaginal symptoms score, we included the item “vaginal dryness” from the corresponding BR45 score (originally called endocrine sexual symptoms), because we did not want to omit this commonly reported symptom.

Data on tumor characteristics and treatment were derived from the medical records. Body mass index (BMI) was calculated from weight and height as assessed by study personnel at the baseline visit.

Anxiety and depressive symptoms were assessed with the 4‐item Patient‐Health‐Questionnaire (PHQ‐4). Patients were considered to have elevated levels of depression or of anxiety if they scored ≥3 on the associated 2‐item subscores (scale 0–6).^14^

### 2.4 Statistical analysis

Descriptive statistics were used to characterize the study sample and the baseline BR42 scores (excluding the two enrolled male participants since this manuscript focusses on sexual and vaginal problems). Distributions of the BR42 scores were presented as a bar plot, after reversing the scale where necessary, so that lower scores present poorer functions/more problems, and using the categorization <25: very poor; 25 to <50: poor; 50 to <75: good; ≥75: very good. Linear regression models were used for each BR42 score separately as a dependent continuous variable, using the baseline sample data before randomization. As independent factors in the models, we simultaneously included sociodemographic factors, that is, age (continuous), BMI (<25/25 to 30/≥30), education (low/middle/higher/academic), married/living with a life partner (yes/no); cancer therapy‐related factors, that is, line of treatment (1st or 2nd/higher), time since mBC diagnosis (log‐transformed), chemotherapy (ever/never), endocrine therapy (ever/never), targeted or immune therapy (ever/never), type of breast surgery (breast conserving/mastectomy with immediate construction/other mastectomy/no breast surgery); and psychological factors, that is, anxiety (yes/no) and depression (yes/no) based on PHQ‐4 cutoffs ≥3 according to Kroenke et al. 2009^14^; additionally adjusting for country. For clarity of presentation, we decided on the same model for all BR42 scores, after having checked for model fit and collinearities. The following variables were investigated as well but showed no significant associations with BR42 scores and did not substantially (< 10%) alter the association of other significant predictors when added to the model, and hence were not included in the final models for reasons of parsimony: location of metastases, recurrent vs. de novo disease, time since diagnosis, employment status, menopausal status, emotional distress (rather than depression and anxiety separately).

Intervention effects on the BR42 scores at 3, 6, and 9 months were analyzed according to the intention‐to‐treat principle using linear mixed effects models taking the hierarchical structure of the data into account. The models were adjusted for the baseline value of the outcome and stratification factors (i.e., center and treatment line). The normality assumption for the residuals was met. Between‐group differences with 95% confidence intervals are presented. As analyses were exploratory and not adjusted for multiple testing, these results must not be interpreted as confirmatory. Cohen's standardized effect sizes (ES) were calculated by dividing the adjusted between‐group difference of the post‐intervention means by the pooled standard deviation of the variable at baseline. As suggested by Cohen, ES <0.2 might be interpreted as no difference, 0.2–0.5 as a small difference, 0.5–0.8 as a medium difference, and 0.8 or more as a large difference.

Intervention effects on chemotherapy side‐effects were also investigated in the subgroup who received chemotherapy at baseline (*n* = 90); and on endocrine symptoms in the subgroup who received endocrine therapy at baseline (*n* = 186). Further, effect modification by age (continuous or ≤50/50–60/>60), marital status (partnered/living alone), or depressive symptoms (score </≥3) was explored by including interaction terms in the mixed models followed by subgroup analyses if *p*_interaction_ ≤.10. All statistical analyses were performed with SAS version 9.4.

## 3 RESULTS

The study included 355 women with mBC with a mean (SD) age of 55.4 (11.2) years, of whom 66.8% were married/living with a life partner and 53.2% had an academic education. At baseline, most patients (74.9%) were on their 1st/2nd line of treatment, 57.2% were undergoing targeted or immune therapy, 52.4% were undergoing endocrine therapy, and 25.4% were receiving chemotherapy. Most patients had bone metastases (67.6%) (Table 1). At 3, 6, and 9 months, 301, 283, and 269 participants, respectively, completed the BR42 questionnaire (see reasons for drop‐outs in the flow chart in Figure S1).

**TABLE 1:** Baseline characteristics of the study participants.

	Total	Exercise group	Control group
	(*n* = 355)	(*n* = 177)	(*n* = 178)
Age, mean (SD)	55.4 (11.2)	54.9 (11.6)	55.9 (10.7)
Body mass index, *n* (%)			
<25	170 (47.9)	91 (51.4)	79 (44.4)
≥25 to <30	107 (30.1)	44 (24.9)	63 (35.4)
≥30	78 (22.0)	42 (23.7)	36 (20.2)
Education, *n* (%)			
Low education	12 (3.4)	7 (3.6)	5 (2.8)
Middle education	74 (20.9)	35 (19.8)	39 (21.9)
Higher education	79 (22.3)	36 (20.3)	43 (42.2)
Academic education^a^	189 (53.2)	99 (55.9)	90 (50.6)
Other	1 (0.3)	0 (0.0)	1 (0.6)
Married/living with life partner, *n* (%)			
Yes	237 (66.8)	121 (68.0)	116 (65.2)
No	118 (33.2)	56 (32.0)	62 (34.8)
Months since first BC diagnosis			
Median (Q1–Q3)	66.8 (32.2–128.6)	75.0 (26.4–128.8)	65.3 (33.0–124.3)
Months since mBC diagnosis			
Median (Q1–Q3)	23.0 (8.3–53.8)	23.1 (7.8–54.7)	22.5 (8.8–49.7)
Type of breast surgery			
Breast conserving surgery	104 (29.3)	55 (31.1)	49 (27.5)
Masectomy with immediate construction	43 (12.1)	22 (12.4)	21 (1.8)
Mastectomy, other	90 (25.4)	42 (23.7)	48 (27.0)
None	116 (32.7)	58 (32.8)	58 (32.6)
Missing	2 (0.6)	0 (0.0)	2 (1.1)
Location of metastases, *n* (%)			
Bone metastases	240 (67.6)	116 (65.5)	124 (69.7)
Lung metastases	94 (26.5)	48 (27.1)	46 (25.8)
Liver metastases	124 (34.9)	67 (37.9)	57 (32.0)
Lymph node metastases	136 (38.3)	67 (37.9)	69 (38.8)
Line of treatment, *n* (%)			
1st or 2nd line	266 (74.9)	133 (75.1)	133 (74.7)
3rd or higher	89 (25.1)	44 (24.9)	45 (25.3)
Chemotherapy, *n* (%)			
Ongoing at baseline	90 (25.4)	48 (27.1)	42 (23.6)
Ever	254 (71.6)	123 (69.5)	131 (73.6)
Endocrine treatment, *n* (%)			
Ongoing at baseline	186 (52.4)	93 (52.5)	93 (52.3)
Ever	261 (73.5)	132 (74.6)	129 (72.5)
Targeted/immune therapy, *n* (%)			
Ongoing at baseline	203 (57.2)	104 (58.8)	99 (55.6)
Ever	248 (69.9)	127 (71.6)	121 (68.0)
Anxiety^b^, *n* (%)	77 (21.7)	42 (23.7)	35 (19.7)
Depression^b^, *n* (%)	55 (15.5)	29 (16.4)	26 (14.6)

Abbreviations: BC, breast cancer; mBC, metastatic breast cancer; Q1, Q3: 1st and 3rd quartile.

^a^ Completed college, university or post graduate degree.

^b^ Elevated PHQ‐4 screening scores for anxiety and depression, respectively; based on cutoffs ≥3 according to Kroenke et al. 2009.

### 3.1 Description of reported symptoms at baseline

**FIGURE 1:**
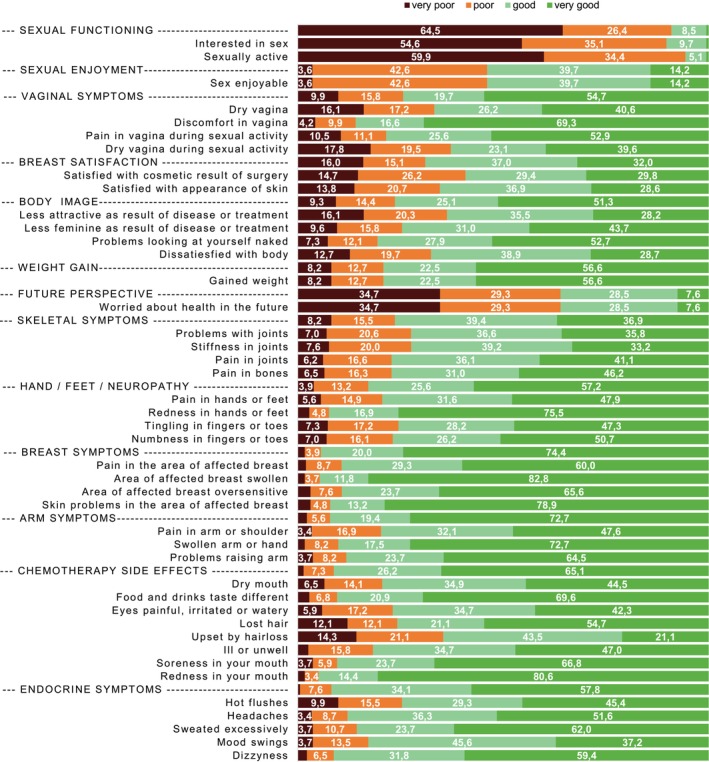
The figure presents the baseline EORTC QLQ‐BR42 function and symptom scores (labeled in capital letters) followed by the corresponding single items. Scores are reversed for this figure, where necessary, so that lower scores present poorer functions/more problems. Function and symptom scores were categorized as <25: very poor; 25 to <50: poor; 50 to <75: good; ≥75: very good.

Figure 1 shows the baseline distribution of the BR42 function and symptom scores (labeled in capital letters) followed by the corresponding single items. Scores are reversed for this figure, where necessary, so that lower scores present poorer functions/more problems. Sexual functioning was (very) poor in the majority of patients (score < 25 in 64.5% and score 25–50 in 26.4% of the women, mean = 17.1, SD = 19.9). More than half of the women were not sexually active at all within the 4 weeks prior to baseline (59.9%), and 54.6% were not at all interested in sex. Among the sexually active women (*n* = 142), 46.2% felt little or no enjoyment. Vaginal problems were also frequent, with 33.3% of women reporting “quite a bit” or “very much” vaginal dryness, in general, and 37.3% during sex.

Among all participants who had undergone breast surgery at any stage, 40.9% reported being “not at all” or “little” satisfied with the cosmetic result and 34.5% with the appearance of the skin. Body image issues were reported relatively frequently, especially feeling less attractive because of the disease or treatment (36.4%) or dissatisfaction with the body (32.4%).

Regarding the BR42 scale “skeletal symptoms,” problems with joints as well as stiffness in the joints were reported by 27.6%. Chemotherapy side effects comprised mainly hair loss (24.2%), painful/irritated eyes (23.1%), and a dry mouth (20.6%). Endocrine symptoms comprised mainly hot flushes (25.4%).

### 3.2 Factors associated with the reported symptoms

Table 2 summarizes the results of the linear regression models regarding the associations of patient characteristics with the BR42 function and symptom scores. Older age and higher levels of depression were associated with worse sexual functioning. Endocrine therapy and higher levels of anxiety were associated with lower sexual enjoyment. Endocrine therapy as well as younger age, having a partner, depressive symptoms, and a longer time since mBC diagnosis (i.e., being longer on mBC treatment) were associated with more vaginal symptoms. Mastectomy, compared to breast conserving surgery, was associated with lower breast satisfaction, especially when there was no immediate breast reconstruction. Body image appeared to be poorer with increasing age, education, anxiety, or depression. Factors associated with skeletal symptoms included obesity and anxiety.

**TABLE 2:** Characteristics associated with the EORTC QLQ‐BR42 function and symptom scores based on linear regression models.

		Sexual functioning			Sexual enjoyment			Vaginal symptoms			Breast satisfaction			Body image			Future perspective		
		*β*	SE	*p*	*β*	SE	*p*	*β*	SE	*p*	*β*	SE	*p*	*β*	SE	*p*	*β*	SE	*p*
Intercept		31.6	8.9	.0004	71.7	20.5	.0007	7.8	12.0	.5191	81.2	18.5	<.0001	47.0	11.3	<.0001	7.4	13.3	.5809
Age	[years]	**−0.3**	**0.1**	.**0024**	−0.1	0.3	.6122	**−0.3**	**0.1**	.**0160**	−0.2	0.2	.3203	**0.5**	**0.1**	.**0002**	**0.4**	**0.2**	.**0052**
Body mass index	<25	0.0			0.0			0.0			0.0			0.0			0.0		
	≥25 to <30	4.5	2.4	.0661	7.3	5.2	.1608	−2.0	3.3	.5371	−0.1	5.0	.9865	−6.0	3.1	.0538	3.3	3.7	.3680
	≥30	0.2	2.8	.9442	2.4	6.2	.6981	−1.0	3.8	.7918	−6.4	5.9	.2830	**−15.4**	**3.5**	**<.0001**	−4.9	4.2	.2416
Education	Academic education	0.0			0.0			0.0			0.0			0.0			0.0		
	Higher education	1.3^b^	2.7	.6482	0.4	5.7	.9418	−3.4	3.7	.3545	6.7	5.6	.2338	**7.1**	**3.5**	.**0414**	4.6	4.1	.2633
	Middle education	0.7	2.9	.8127	5.5	6.2	.3816	−3.7	4.0	.3545	−7.2	6.4	.2599	**8.5**	**3.7**	.**0243**	0.4	4.4	.9241
	Low education	−7.7	6.3	.2245	7.3	20.1	.7172	−15.8	8.6	.0662	**28.7**	**12.0**	.**0180**	**25.3**	**8.1**	.**0019**	1.1	9.5	.9045
Married/partnered	Yes vs. no	2.7	2.3	.2426	−2.9	5.8	.6230	**14.9**	**3.1**	**<.0001**	2.5	4.7	.5859	3.7	2.9	.2094	−1.8	3.5	.6113
Line of treatment	1st/2nd vs. higher	0.7	2.6	.8001	−4.7	6.0	.4337	2.5	3.6	.4886	3.1	5.1	.5516	4.3	3.3	.2035	**9.3**	**3.9**	.**0196**
Time since mBC diagnosis	[months, log‐transformed]	−0.6	0.9	.5325	0.2	2.0	.9193	**2.6**	**1.2**	.**0289**	2.1	1.9	.2772	0.4	1.1	.7321	1.8	1.3	.1796
Chemotherapy	Ever vs. never	−2.4	2.6	.3400	−7.0	5.5	.2064	6.4	3.5	.0650	0.7	5.5	.8992	−5.7	3.3	.0819	−0.9	3.8	.8129
Endocrine therapy	Ever vs. never	1.2	2.7	.6481	−12.9	6.8	.0613	**9.5**	**3.7**	.**0102**	−5.6	5.9	.3456	−0.0	3.5	.9912	6.2	4.1	.1288
Targeted/immune therapy	Ever vs. never	1.2	2.5	.6302	7.0	5.7	.2218	3.9	3.4	.2614	−2.5	5.1	.6184	2.2	3.2	.4986	−0.7	3.8	.8594
Breast surgery	Breast conserving surgery	0.0			0.0			0.0			0.0			0.0			0.0		
	Mastectomy with immediate construction	4.2	3.6	.2480	6.5	7.3	.3749	−8.7	4.9	.0777	−10.7	6.2	.0847	−1.4	4.6	.7688	3.5	5.5	.5169
	Mastectomy, other	−0.5	2.9	.8631	−0.3	6.7	.9653	−4.7	3.9	.2357	**−14.2**	**5.1**	.**0057**	0.2	3.7	.9476	3.6	4.4	.4036
	None	8.5	14.3	.5501	29.3	27.0	.2787	13.6	19.5	.4852	n.a.			−18.8	18.3	.3041	−25.1	21.5	.2443
Anxiety^a^	Yes vs. no	1.7	2.9	.5570	−10.2	6.2	.1063	3.0	3.9	.4485	−3.3	6.1	.5884	**−7.3**	**3.7**	.**0481**	**−15.4**	**4.3**	.**0005**
Depression^a^	Yes vs. no	**−10.3**	**3.4**	.**0025**	−5.3	8.9	.5488	**9.6**	**4.6**	.**0368**	−5.2	7.1	.4632	**−19.0**	**4.3**	**<.0001**	**−18.3**	**5.1**	.**0004**
Country	Netherlands	0.0			0.0			0.0			0.0			0.0			0.0		
	Australia	−2.7	4.0	.5007	−4.7	9.7	.6310	3.9	5.4	.4719	−13.6	7.4	.0677	**−10.8**	**5.1**	.**0342**	−11.2	6.0	.0625
	Germany	**8.2**	**3.1**	.**0081**	11.7	6.5	.0735	**17.7**	**4.2**	**<.0001**	11.9	6.4	.0661	**−11.8**	**3.9**	.**0030**	**−13.7**	**4.7**	.**0036**
	Poland	−1.2	4.0	.7709	5.6	8.6	.5163	**13.2**	**5.4**	.**0150**	**−25.2**	**8.3**	.**0026**	−4.4	5.0	.3855	−7.9	6.0	.1857
	Spain	−0.1	3.7	.9822	−6.9	7.5	.3625	7.7	5.0	.1261	−14.7	7.9	.0645	−7.1	4.7	.1280	−6.4	5.5	.2448
	Sweden	−3.0	4.1	.4601	−3.6	10.3	.7290	**13.9**	**5.5**	.**0111**	**−16.1**	**7.9**	.**0443**	−7.7	5.1	.1351	1.9	6.0	.7560

		Chemotherapy side effects			Endocrine symptoms			Hand/foot, neuropathy			Skeletal symptoms			Weight gain			Arm symptoms			Breast symptoms		
		*β*	SE	*p*	*β*	SE	*p*	*β*	SE	*p*	*β*	SE	*p*	*β*	SE	*p*	*β*	SE	*p*	*β*	SE	*p*
Intercept		17.3	7.4	.0201	44.5	7.2	<.0001	−4.5	10.0	.6533	28.7	11.6	.0139	39.8	14.0	.0048	18.5	8.7	.0328	17.6	7.4	.0172
Age	[years]	−0.0	0.1	.9842	**−0.5**	**0.1**	**<.0001**	**0.2**	**0.1**	.**0424**	−0.0	0.1	.8302	**−0.6**	**0.2**	.**0001**	−0.0	0.1	.9251	0.0	0.1	.9473
Body mass index	<25	0.0			0.0			0.0			0.0			0.0			0.0			0.0		
	≥25 to <30	0.5	2.0	.8066	1.7	2.0	.3924	**7.5**	**2.8**	.**0067**	4.8	3.2	.1351	**15.6**	**3.9**	**<.0001**	**5.2**	**2.4**	.**0312**	**4.7**	**2.0**	.**0201**
	≥30	0.9	2.3	.6949	3.4	2.3	.1301	**7.3**	**3.1**	.**0203**	**11.3**	**3.6**	.**0020**	**18.8**	**4.4**	**<.0001**	**6.6**	**2.7**	.**0149**	**6.8**	**2.3**	.**0035**
Education	Academic education	0.0			0.0			0.0			0.0			0.0			0.0			0.0		
	Higher education	−0.7	2.3	.7614	0.2	2.2	.9249	−2.2	3.1	.4795	−0.5	3.6	.8783	−1.7	4.3	.7014	−1.0	2.7	.7128	0.8	2.3	.7267
	Middle education	1.7	2.4	.4943	3.4	2.4	.1612	5.3	3.3	.1076	3.2	3.8	.4103	6.1	4.6	.1909	**7.4**	**2.9**	.**0097**	**5.5**	**2.4**	.**0235**
	No or basic education	−7.8	5.3	.1405	−8.5	5.1	.0991	−4.8	7.1	.5006	0.6	8.3	.9406	−0.8	10.0	.9373	0.9	6.2	.8817	−0.7	5.2	.8963
Married/partnered	Yes vs. no	−0.5	1.9	.7909	**−5.7**	**1.9**	.**0023**	−2.3	2.6	.3815	−4.4	3.0	.1490	1.4	3.6	.7077	**−4.8**	**2.2**	.**0323**	−2.7	1.9	.1550
Line of treatment	1st/2nd vs. higher	**−4.5**	**2.2**	.**0399**	−1.4	2.1	.5217	−3.5	3.0	.2445	−5.1	3.4	.1396	**11.6**	**4.2**	.**0055**	−4.6	2.6	.0753	−2.3	2.2	.2937
Time since mBC diagnosis	[months, log‐transformed]	−0.9	0.7	.2327	−0.3	0.7	.7004	1.3	1.0	.2099	0.3	1.2	.7929	2.1	1.4	.1299	−0.2	0.9	.7738	−1.3	0.7	.0853
Chemotherapy	Ever vs. never	**4.9**	**2.1**	.**0217**	−1.6	2.1	.4551	**10.0**	**2.9**	.**0006**	−2.5	3.3	.4549	−3.7	4.0	.3607	2.8	2.5	.2617	1.0	2.1	.6228
Endocrine therapy	Ever vs. never	1.3	2.3	.5752	**7.0**	**2.2**	.**0016**	−0.3	3.1	.9288	5.6	3.5	.1160	2.0	4.3	.6344	−1.9	2.6	.4693	**−5.1**	**2.2**	.**0247**
Targeted/immune therapy	Ever vs. never	−1.4	2.1	.5126	−0.9	2.1	.6625	−3.8	2.9	.1824	−0.1	3.3	.9754	−6.9	4.0	.0859	1.6	2.5	.5105	−0.9	2.1	.6694
Breast surgery	Breast conserving surgery	0.0			0.0			0.0			0.0			0.0			0.0			0.0		
	Mastectomy with immediate construction	**−6.6**	**3.0**	.**0288**	−2.4	3.0	.4127	−4.5	4.1	.2714	−1.3	4.7	.7908	−1.5	5.7	.7887	−1.8	3.5	.6151	−3.6	3.0	.2324
	Mastectomy, other	−0.2	2.4	.9438	−1.1	2.4	.6447	−1.8	3.3	.5885	0.7	3.8	.8577	0.9	4.6	.8464	0.5	2.8	.8657	0.9	2.4	.7037
	None	−3.0	2.4	.2091	−4.4	2.3	.0577	**−7.3**	**3.2**	.**0230**	−6.8	3.7	.0677	**−10.8**	**4.5**	.**0161**	**−6.6**	**2.8**	.**0172**	**−5.1**	**2.3**	.**0308**
Anxiety^a^	Yes vs. no	1.6	2.4	.5046	**7.5**	**2.3**	.**0016**	2.3	3.3	.4889	**8.4**	**3.8**	.**0275**	**9.2**	**4.6**	.**0441**	4.6	2.8	.1000	**5.3**	**2.4**	.**0289**
Depression^a^	Yes vs. no	**8.2**	**2.8**	.**0038**	4.3	2.7	.1168	2.7	3.8	.4725	4.5	4.4	.3138	1.4	5.3	.7962	1.4	3.3	.6660	1.9	2.8	.5095
Country	Netherlands	0.0			0.0			0.0			0.0			0.0			0.0			0.0		
	Australia	6.0	3.3	.0731	0.6	3.3	.8459	6.2	4.5	.1684	−0.4	5.2	.9317	−9.4	6.3	.1389	−1.5	3.9	.6922	−1.8	3.3	.5905
	Germany	**15.3**	**2.6**	**<.0001**	**9.6**	**2.5**	.**0002**	**16.6**	**3.5**	**<.0001**	3.4	4.1	.4015	2.8	4.9	.5615	**10.7**	**3.0**	.**0005**	**10.5**	**2.6**	**<.0001**
	Poland	**8.2**	**3.3**	.**0138**	**8.0**	**3.2**	.**0141**	**14.1**	**4.5**	.**0017**	7.6	5.2	.1458	−7.2	6.3	.2500	5.0	3.9	.1965	**9.2**	**3.3**	.**0055**
	Spain	**9.2**	**3.1**	.**0029**	3.0	3.0	.3147	**8.6**	**4.1**	.**0386**	0.2	4.8	.9677	7.3	5.8	.2067	−4.0	3.6	.2629	−0.9	3.0	.7590
	Sweden	5.9	3.4	.0809	4.6	3.3	.1634	**9.9**	**4.5**	.**0304**	4.6	5.3	.3868	1.8	6.4	.7824	−1.8	3.9	.6443	0.2	3.3	.9552

^a^ Elevated PHQ‐4 screening scores for anxiety and depression, respectively; based on cutoffs ≥3 according to Kroenke et al. 2009.

^b^ The bold values indicates p < .05.

### 3.3 Effects of exercise

Table 3 presents the results of the linear mixed effects models investigating the exercise intervention effect on the BR42 function and symptom scores. At 6 months, IG reported better sexual functioning compared to CG (between‐group difference (BGD) with 95%CI = 5.6 [1.9, 9.4], ES = 0.28), which still persisted at 9 months (BGD: 4.5 [0.7, 8.3], ES = 0.23). At all time points, sexual enjoyment (if sexually active) was improved in IG compared to CG, albeit not statistically significantly, probably due to the small subsample size (BGD: 8.3 [−0.0, 16.6], ES = 0.32 at 9‐month). Further, there was a significant exercise effect on vaginal symptoms at 6 months (BGD: −7.1 [−11.7, −2.5], ES = 0.25), which was attenuated at 9 months (BGD: −4.3 [−9.0, 0.4], ES = 0.15).

**TABLE 3:** Effects of the PREFERABLE‐EFFECT exercise program on EORTC QLQ‐BR42 function and symptom scores.

		Between‐group differences					
		At 3 months		At 6 months		At 9 months	
		Mean (95% CI)	ES	Mean (95% CI)	ES	Mean (95% CI)	ES
Sexual functioning	IG	3.2 (−0.4, 6.8)	0.16	**5.6 (1.9, 9.4)** **	0.28	**4.5 (0.7, 8.3)** *	0.23
	CG	Reference		Reference		Reference	
Sexual enjoyment	IG	4.7 (−2.7, 12.0)	0.18	4.9 (−2.9, 12.7)	0.19	8.3 (−0.0, 16.6)	0.32
	CG	Reference		Reference		Reference	
Vaginal symptoms	IG	−2.8 (−7.2, 1.7)	0.10	**−7.1 (−11.7, −2.5)** **	0.25	−4.3 (−9.0, 0.4)	0.15
	CG	Reference		Reference		Reference	
Breast satisfaction	IG	**7.5 (0.4, 14.6)** *	0.23	3.3 (−4.0, 10.6)	0.10	2.0 (−5.4, 9.4)	0.06
	CG	Reference		Reference		Reference	
Body Image	IG	3.7 (−0.4, 7.9)	0.13	4.0 (−0.2, 8.3)	0.14	2.8 (−1.6, 7.1)	0.10
	CG	Reference		Reference		Reference	
Future perspective	IG	−0.3 (−6.1, 5.5)	0.01	3.2 (−2.7, 9.2)	0.10	0.1 (−6.0, 6.1)	0.00
	CG	Reference		Reference		Reference	
Chemotherapy side effects	IG	−2.3 (−5.4, 0.8)	0.13	−3.1 (−6.2, 0.1)	0.18	**−4.7 (−7.9, −1.5)** **	0.27
	CG	Reference		Reference		Reference	
Endocrine symptoms	IG	−1.5 (−4.6, 1.5)	0.09	−0.2 (−3.4, 3.0)	0.01	−0.2 (−3.5, 3.0)	0.01
	CG	Reference		Reference		Reference	
Hand/foot/neuropathy	CG	1.8 (−2.6, 6.1)	0.08	−0.7 (−5.1, 3.8)	0.03	−2.1 (−6.7, 2.4)	0.09
	IG	Reference		Reference		Reference	
Arm symptoms	IG	0.4 (−3.5, 4.2)	0.02	−0.9 (−4.8, 3.1)	0.04	−0.9 (−4.9, 3.1)	0.04
	CG	Reference		Reference		Reference	
Breast symptoms	IG	−1.1 (−4.0, 1.8)	0.06	−2.4 (−5.3, 0.6)	0.14	−0.1 (−3.1, 2.9)	0.01
	CG	Reference		Reference		Reference	
Skeletal symptoms	IG	−2.7 (−7.7, 2.3)	0.10	−0.6 (−5.7, 4.4)	0.02	−3.0 (−8.1, 2.2)	0.12
	CG	Reference		Reference		Reference	
Weight gain	IG	−4.0 (−9.3, 1.3)	0.12	1.9 (−3.6, 7.3)	0.06	−1.9 (−7.5, 3.6)	0.06
	CG	Reference		Reference		Reference	

*Note*: Models were adjusted for the baseline value of the outcome and stratification factors (center and therapy line). Significant between‐group differences are in bold.

Abbreviations: CG: control group; ES: effect size; IG: intervention group.

* *p* < .05;

** *p* < .01.

At 3 months, breast satisfaction was improved in IG compared to CG (BGD: 7.5 [0.4, 14.6], ES = 0.23, Table 3); however, the effects were attenuated at 6 and 9 months. Effects on body image were only marginally in favor of IG (BGD: 4.0 [−0.2, 8.3], ES = 0.14 at 6‐month). IG reported fewer chemotherapy side effects at 6 months (BGD: −3.1 [−6.2, 0.1], ES = 0.18) and 9 months (BGD: −4.7 [−7.9, −1.5], ES = 0.27) compared to CG.

Regarding the subgroup of patients undergoing also chemotherapy at baseline (Table S1), the data indicated significantly fewer chemotherapy side‐effects in IG compared to CG at 3, 6, and 9 months (BGD −7.0 [−14.0, −0.1], ES = 0.41; −8.2 (−15.4, −1.0), ES = 0.48; −10.0 (−17.5, −2.6), ES = 0.58; respectively). In contrast, exercise did not result in fewer endocrine symptoms, neither in the whole group nor in the subgroup on endocrine treatment at baseline.

Effect modification analyses highlighted the effect of exercise on vaginal symptoms (*p*_interaction_ = .069) and skeletal symptoms (*p*_interaction_ = .072) by age subgroups (Table S2). Results suggested a stronger exercise effect on vaginal symptoms in women aged ≤50 years than in women aged 50–60 or >60 years. Similarly, there was a stronger effect on sexual enjoyment (when sexually active) in the younger group, although the interaction term was not statistically significant (*p*_interaction_ = .45), likely due to the small sample size for this variable. Skeletal symptoms were significantly reduced in IG compared to CG at 3, 6, and 9 months (BGD: −9.9 [−18.7, −1.2]; −12.3 (−21.3, −3.3); −10.8 (−19.8, −1.9), respectively) among patients above the age of 60. In contrast, there were no indications of an exercise effect on the skeletal symptoms in patients aged 50–60 or younger than 50 years of age (Table S2).

## 4 DISCUSSION

Our analyses of the PREFERABLE‐EFFECT study indicate that women with mBC have low sexual functioning or little sexual enjoyment and often suffer from vaginal symptoms. Many participants also reported an impaired body image, dissatisfaction with their breasts, skeletal symptoms, chemotherapy side effects (mainly hair loss, dry mouth, painful or irritated eyes), or hot flushes. The results demonstrated that a supervised exercise program can help women with mBC to maintain or improve sexual functioning and enjoyment, and to reduce vaginal symptoms, and in specific subgroups also skeletal symptoms and chemotherapy side effects.

The women in this study reported low sexual functioning, that is, the majority had no or little sexual activity (with and without intercourse) or interest in sexual activities, which decreased with age and level of depression. With an average score of 17.1, sexual functioning among women with mBC seems to be lower than among women with non‐metastatic breast cancer, where other studies using the BR23/45 have found mean scores of 26.4 to 29.1.^15^, ^16^, ^17^ Patients with (non‐metastatic) breast cancer, in‐turn, have been shown to have more sexual problems than women in the general female population.^18^, ^19^, ^20^, ^21^ Moreover, of the sexually active patients in our study, almost half indicated experiencing little or no sexual enjoyment. Sexual interest, activity, and enjoyment can be related to desire, arousal, lubrication, orgasm, satisfaction, and pain. Reasons for impairment might include somatic problems such as vaginal dryness or dyspareunia, psychological issues such as stress or depression due to the life‐threatening disease, impaired body image, or reduced breast and nipple sensation due to surgery or radiation therapy.^4^ Reporting of the item “vaginal pain during sexual activity” might sometimes also relate to vaginismus, an involuntary contraction of the musculature of the vagina, which interferes with sexual intercourse.^22^ The consequences of mBC might also have a negative impact on the relationship as well as the partner's interest in sex, which in turn may lead to reduced sexual activity or enjoyment of the patient.^23^

One third of our patients with mBC reported high levels of vaginal dryness and a quarter reported vaginal pain during sexual activity. Vaginal symptoms were associated with endocrine therapy and younger age. Vaginal dryness is a frequent problem in patients with mBC as well as in survivors of non‐metastatic breast cancer undergoing endocrine therapy.^4^, ^24^, ^25^, ^26^ The most common cause of vaginal dryness is hormonal change associated with endocrine therapy or (therapy‐induced) menopause. Estrogens play a crucial role in maintaining the thickness and elasticity of the vaginal lining and promoting natural lubrication.^27^ Psychological factors, such as stress and anxiety, affect overall hormonal balance in the body and may also contribute to vaginal dryness. Vaginal estrogen therapy can help to revive vaginal tissues. However, a recent study showed that (estriol‐based) vaginal estrogen therapy reduced disease‐free survival in BC patients using aromatase inhibitors.^28^ Based on our results, exercise could be considered a potentially beneficial approach to addressing sexual dysfunction or vaginal problems in cancer patients.

In a study of mBC, participants emphasized the importance of sexual activities with their partners, but expressed concerns about physical limitations, including frequent bone and vaginal pain during intercourse.^29^ However, the women's health care providers often concentrated solely on recommending vaginal lubricants, overlooking the broader spectrum of women's issues. The European Society for Medical Oncology (ESMO) recommends that women with breast cancer who have chemotherapy‐induced amenorrhea, receive endocrine therapy, or undergo ovarian function suppression should receive appropriate counseling regarding sexual functioning.^30^ The ESMO guideline mentions non‐hormonal therapies, for example, vaginal moisturizers, lubricants, and gels, as first‐choice treatment. In patients where these measures do not help, they suggest consideration of limited and selective use of hormonal agents.^30^ Recommendations of the American Cancer Society/American Society of Clinical Oncology are similar.^31^ So far, exercise has not been considered a treatment option, probably due to limited studies. Indeed, the American College of Sports Medicine (ACSM) Roundtable recently rated the evidence regarding exercise effects on sexual functioning in cancer survivors as insufficient.^6^, ^11^

To‐date, randomized controlled trials (RCTs) investigating exercise effects on sexual functioning in women (with or without cancer) are scarce and have mainly investigated pelvic floor exercise.^9^ We are aware of only two RCTs investigating the effect of some other types of exercise on female cancer patients' sexual health. The first RCT randomized 47 BC survivors to a 16‐week Pilates program, belly dance, or a control group, and found no significant group differences in sexual function overall, but a significant improvement in pain/discomfort only for the belly dance group.^32^ The second study, a 4‐arm RCT in 422 non‐metastatic BC patients 0.5–5 years after completion of chemotherapy, found significant improvements in the groups that received cognitive behavioral therapy (CBT) alone or in combination with a 12‐week self‐directed exercise program (2.5–3 h/week, swimming, running, cycling, etc.), but not in the group that received the 12‐week exercise program alone without CBT.^10^ In contrast, our supervised combined aerobic and resistance exercise intervention significantly improved sexual functioning at the primary 6‐month endpoint, and the effects were sustained at 9 months. This difference from the previous RCT might be due to the more advanced disease of our study sample, or to our use of supervised moderate‐to‐high intensity exercise including resistance, aerobic, and balance training with a longer intervention duration. While the effect sizes we observed were small‐to‐moderate, they are comparable to the magnitude of exercise effects typically observed on quality of life outcomes.^33^ Likewise, our results showing slight improvement in vaginal symptoms (which comprised mainly vaginal dryness overall and during intercourse) in IG versus CG suggest that exercise may also help alleviate the discomfort caused by vaginal dryness. Effects of exercise on vaginal symptoms were stronger among women below the age of 50, who may have experienced therapy‐induced menopause. Likewise, younger women in our study seemed to have benefited more from the exercise intervention in terms of sexual enjoyment.

Various biological pathways for the effects of exercise on sexual functioning, enjoyment, and vaginal symptoms have been proposed in the literature.^8^ For example, acute exercise effects (i.e., immediately following an exercise session) on physiological sexual arousal seem to be driven by increases in sympathetic nervous system activity and endocrine factors. Longer‐term effects of exercise on sexual functioning might be related to improved cardiovascular health, as hypertension and impaired vascular function are associated with female sexual dysfunction. An improved body image has also been posited as a mediator of the effect of exercise on sexual functioning, but this could not be confirmed with our data. Vaginal dryness can be the result of impaired function of the vaginal epithelium due to reduced vaginal blood flow, thinning of the mucosa, changes in the microbiome, or inflammation.^34^ A major triggering factor is a reduced estrogen level. Pathways that elucidate how exercise may influence vaginal dryness are not yet well studied. It might be that beneficial effects on blood flow and inflammation could play a role.

Our mBC sample reported poorer body image (mean score: 66.2) than women with non‐metastatic BC (reported mean scores ranging from 76.9 to 92.0).^15^, ^16^, ^17^ Our study showed only a weak effect of the exercise intervention on body image. Similarly, in a scoping review, only one of five identified RCTs investigating the effect of resistance exercise training in BC survivors showed a significant effect on body image.^35^, ^36^ Thus, for improvement of body image, an exercise intervention alone might not be sufficient. A recent meta‐analysis including six RCTs with a total of 758 BC patients demonstrated that cognitive‐based interventions significantly reduced negative body image perception.^38^

More than one quarter of study participants suffered from skeletal symptoms, such as stiffness in joints or pain in joints or bones. Arthralgia, which includes joint pain and stiffness, is a well‐known problem in post‐menopausal women on aromatase inhibitors, especially in obese patients.^37^, ^39^ We also found that obesity was significantly associated with higher levels of skeletal symptoms. Moreover, in our study, an exercise effect on skeletal symptoms was seen among patients above the age of 60, but not in younger patients. As aromatase inhibitors are more frequently used as endocrine therapy in women above age 60 years compared to younger women, the observed exercise effect on skeletal symptoms might be attributed to mitigation of aromatase inhibitor induced arthralgia, as has been suggested by previous studies.^40^ However, due to limited subgroup sample size, we cannot rule out the possibility that this is a chance finding, so further research is needed.

We also observed an effect of exercise on chemotherapy‐related side‐effects as assessed by the BR42, and specifically on painful, watering, or irritated eyes, dry or sore mouth, and feeling unwell. We are not aware of any previous RCTs that investigated exercise effects on chemotherapy‐induced eye or mouth disorders, and the underlying mechanisms are unclear. Further research is needed to better understand the potential impact of exercise on these symptoms.

As limitation of the study should be noted that patients were able to participate in the study at any time during their treatment, which resulted in a heterogeneous sample but also increases the generalizability of our results. Moreover, only the frequency or severity of symptoms were assessed but not the perceived burden. However, in a study in breast cancer survivors undergoing endocrine therapy, the majority of sexually active women who reported sexual dysfunction (134 of 142, 94%) also reported having distress by at least one sexual impairment, which was considered by the authors as clinically relevant sexual dysfunction.^25^ Further, all models have been adjusted for country for which also some significant associations were seen. However, findings on country differences need to be interpreted cautiously because in the EORTC questionnaire version that was valid at the time of study initiation the German translation of the Likert scale category “quite a bit” did not fully match the meaning in the other languages.^41^ Finally, these analyses were exploratory in nature and were not adjusted for multiple testing. Hence, we cannot exclude the possibility of some chance finding. We would recommend that additional research be undertaken to replicate our findings. As strength can be noted that this multinational RCT is so‐far the largest study investigating the effectiveness of exercise on sexual functioning, vaginal symptoms, body image and other symptoms in patients with mBC.

In summary, our study showed that many patients with mBC experience sexual health problems or vaginal symptoms, have an impaired body image, skeletal symptoms, and other therapy‐related side effects. By shedding light on this under‐researched area, we hope to raise awareness among healthcare professionals of the problems faced by patients with mBC and encourage further exploration. Our results demonstrated that a supervised, structured exercise program can help women with mBC to maintain or improve sexual functioning and enjoyment, reduce vaginal symptoms, and in specific subgroups also skeletal symptoms and chemotherapy‐related irritations of the eyes or mouth. Therefore, the integration of exercise as a supportive care strategy should be promoted, not only to increase health‐related quality of life and reduce fatigue, but also to alleviate vaginal symptoms and improve sexual health among women with mBC.

## AUTHOR CONTRIBUTIONS

**Martina E. Schmidt:** Conceptualization; data curation; formal analysis; visualization; writing – original draft; writing – review and editing; funding acquisition; investigation. **Anouk E. Hiensch:** Data curation; investigation; writing – review and editing. **Johanna Depenbusch:** Data curation; investigation; writing – review and editing. **Evelyn M. Monninkhof:** Investigation; writing – review and editing. **Jon Belloso:** Conceptualization; funding acquisition; investigation; writing – review and editing. **Dorothea Clauss:** Investigation; writing – review and editing. **Nadira Gunasekara:** Investigation; writing – review and editing. **Mark Trevaskis:** Investigation; writing – review and editing. **Helene Rundqvist:** Conceptualization; writing – review and editing; investigation; funding acquisition. **Joachim Wiskemann:** Writing – review and editing; supervision; investigation. **Jana Müller:** Investigation; writing – review and editing. **Maike G. Sweegers:** Investigation; writing – review and editing. **Andreas Schneweiss:** Writing – review and editing; investigation. **Renske Altena:** Investigation; writing – review and editing. **Joanna Kufel‐Grabwska:** Writing – review and editing; investigation. **Rhodé M. Bijlsma:** Writing – review and editing; investigation. **Lobke van Leeuwen‐Snoeks:** Investigation; writing – review and editing. **Daan ten Bokkel Huinink:** Investigation; writing – review and editing. **Gabe Sonke:** Investigation; writing – review and editing. **Susanne Brandner:** Investigation; writing – review and editing. **Peter Savas:** Investigation; writing – review and editing. **Yoland Antill:** Investigation; writing – review and editing. **Michelle White:** Investigation; writing – review and editing. **Nerea Ancizar:** Investigation; writing – review and editing. **Elsken van der Wall:** Conceptualization; funding acquisition; writing – review and editing; investigation. **Neil K. Aaronson:** Conceptualization; investigation; funding acquisition; writing – review and editing. **Elzbieta Senkus:** Conceptualization; funding acquisition; writing – review and editing; investigation; supervision. **Ander Urruticoechea:** Conceptualization; funding acquisition; writing – review and editing; supervision. **Eva M. Zopf:** Conceptualization; funding acquisition; writing – review and editing; investigation; supervision. **Wilhelm Bloch:** Conceptualization; funding acquisition; writing – review and editing; investigation; supervision. **Martijn M. Stuiver:** Writing – review and editing; conceptualization; investigation; funding acquisition; supervision. **Yvonne Wengström:** Conceptualization; funding acquisition; writing – review and editing; investigation; supervision. **Anne M. May:** Conceptualization; funding acquisition; writing – review and editing; project administration; supervision; investigation. **Karen Steindorf:** Conceptualization; funding acquisition; supervision; writing – original draft; writing – review and editing; investigation; project administration.

## FUNDING INFORMATION

This study received funding from the European Union's Horizon 2020 research and innovation program (No. 825677) and the National Health and Medical Research Council of Australia (2018/GNT1170698). The funders of the study (the European Union [Horizon 2020] and the National Health and Medical Research Council of Australia) had no role in study design, data collection, data analysis, data interpretation, or writing of the report.

## CONFLICT OF INTEREST STATEMENT

The authors declare no conflicts of interest.

## ETHICS STATEMENT

All patients provided written informed consent. The study was approved by the institutional review board of the University Medical Center Utrecht (19‐524/M) and local ethical review boards, conducted in accordance with Good Clinical Practice and the Declaration of Helsinki, and registered with ClinicalTrials.gov (NCT04120298).

## Supporting information


Data S1.

## Data Availability

Researchers can request current data from PREFERABLE's principal investigator (a.m.may@umcutrecht.nl). Pseudonymized data (including data dictionaries) will be made available through the Digital Research Environment, which is a trusted digital research environment that can be accessed at https://mydre.org. Further information is available from the corresponding author upon request.
