# Supplementary material for: Impact of exercise on sexual health, body image, and therapy‐related symptoms in women with metastatic breast cancer: The randomized controlled PREFERABLE‐EFFECT trial
Source: Int J Cancer. 2025 Apr 3;157(3):490–503. doi: 10.1002/ijc.35429 (PMC12141986; doi:10.1002/ijc.35429)
Supplement: Supplementary file 1 — Data S1. [file IJC-157-490-s001.pdf]

Impact of Exercise on Sexual Health, Body Image, and Therapy-related Symptoms in Women with Metastatic Breast Cancer: The Randomized Controlled PREFERABLE-EFFECT Trial

Martina E. Schmidt, Anouk E. Hiensch, Johanna Depenbusch, Evelyn M. Monninkhof, Jon Belloso, Dorothea Clauss, Nadira Gunasekara, Mark Trevaskis, Helene Rundqvist, Joachim Wiskemann, Jana Müller, Maike G. Sweegers, Andreas Schneeweiss, Renske Altena, Joanna Kufel-Grabwska, Rhodé M. Bijlsma, Lobke van Leeuwen-Snoeks, Daan ten Bokkel Huinink, Gabe Sonke, Susanne Brandner, Peter Savas, Yoland Antill, Michelle White, Nerea Ancizar, Elsken van der Wall, Neil K. Aaronson, Elzbieta Senkus, Ander Urruticoechea, Eva M. Zopf, Wilhelm Bloch, Martijn M. Stuiver, Yvonne Wengstrom, Anne M. May, Karen Steindorf

Supplementary Material

Table of contents

Figure S1: Flow chart

Table S1: Effects of the PREFERABLE-EFFECT exercise program in subgroups by therapy

Table S2: Effects of the PREFERABLE-EFFECT exercise program in subgroups of patients by age

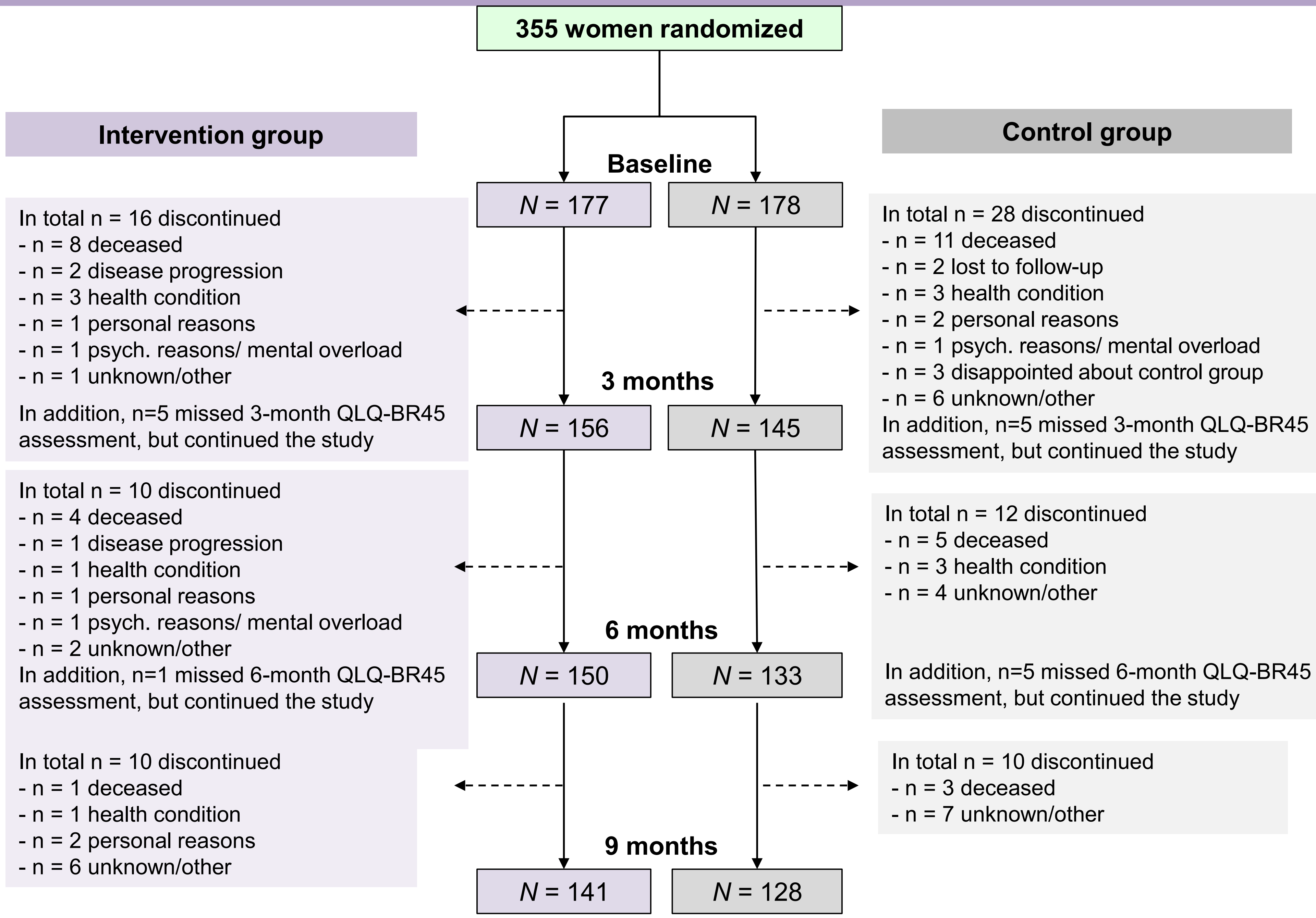

Figure S1: Flow Chart

Supplementary Table S1: Effects of the PREFERABLE-EFFECT exercise program in subgroups by therapy

		Change from Baseline to 3 months			Change from Baseline to 6 months			Change from Baseline to 9 months		
**BR45 Scale**		**Within-group differences**	**Between-group differences**	**ES**	**Within-group differences**	**Between-group differences**	**ES**	**Within-group differences**	**Between-group differences**	**ES**
**Patients undergoing chemotherapy at baseline**										
Sexual function	IG	2.3 (-3.2, 7.9)	6.9 (-0.9, 14.6)	0.34	3.7 (-1.9, 9.3)	8.5 (0.3, 16.6)*	0.42	2.5 (-3.3, 8.3)	6.0 (-2.5, 14.5)	0.30
	CG	-4.5 (-10.2, 1.2)	Reference		-4.8 (-10.9, 1.3)	Reference		-3.5 (-10.0, 3.0)	Reference	
Sexual enjoyment	IG	19.1 (7.6, 30.7)**	12.8 (-5.1, 30.8)	0.50	12.8 (1.7, 24.0)*	13.1 (-5.8, 31.9)	0.51	16.4 (4.8, 28.0)**	14.1 (-5.5, 33.7)	0.55
	CG	6.3 (-7.7, 20.3)	Reference		-0.3 (-15.2, 14.7)	Reference		2.3 (-13.3, 17.9)	Reference	
Vaginal symptoms	IG	0.9 (-6.0, 7.8)	0.0 (-9.7, 9.7)	0.00	-3.8 (-10.7, 3.2)	-6.6 (-16.7, 3.4)	0.23	0.2 (-7.0, 7.4)	-1.9 (-12.4, 8.6)	0.07
	CG	0.9 (-6.2, 8.0)	Reference		2.9 (-4.7, 10.4)	Reference		2.1 (-5.9, 10.0)	Reference	
Chemotherapy side-effects	IG	-6.6 (-11.5, -1.6)*	-7.0 (-14.0, -0.1)*	0.41	-7.9 (-12.9, -2.9)**	-8.2 (-15.4, -1.0)*	0.48	-8.3 (-13.4, -3.1)**	-10.0 (-17.5, -2.6)**	0.58
	CG	0.5 (-4.6, 5.6)	Reference		0.3 (-5.1, 5.6)	Reference		1.7 (-3.8, 7.3)	Reference	
Hand/feet/neuropathy	IG	-1.1 (-8.8, 6.6)	0.9 (-9.9, 11.7)	0.04	-0.8 (-8.6, 6.9)	-4.5 (-15.6, 6.7)	0.19	2.1 (-5.9, 10.1)	0.1 (-11.5, 11.7)	0.01
	CG	-2.0 (-9.9, 6.0)	Reference		3.6 (-4.7, 11.9)	Reference		2.0 (-6.7, 10.7)	Reference	
**Patients undergoing endocrine therapy at baseline**										
Sexual function	IG	3.0 (-1.3, 7.3)	4.7 (-0.4, 9.7)	0.23	0.7 (-3.7, 5.1)	4.3 (-1.0, 9.6)	0.21	-0.2 (-4.6, 4.2)	5.1 (-0.2, 10.4)	0.26
	CG	-1.7 (-6.1, 2.7)	Reference		-3.6 (-8.2, 1.0)	Reference		-5.3 (-10.0, -0.7)*	Reference	
Sexual enjoyment	IG	2.2 (-6.8, 11.1)	2.4 (-7.8, 12.6)	0.09	4.0 (-5.4, 13.5)	4.8 (-5.9, 15.5)	0.19	7.1 (-2.7, 16.9)	8.8 (-2.9, 20.5)	0.35
	CG	-0.2 (-9.6, 9.1)	Reference		-0.8 (-10.3, 8.8)	Reference		-1.7 (-11.8, 8.4)	Reference	
Vaginal symptoms	IG	-1.5 (-6.8, 3.8)	-3.8 (-10.0, 2.4)	0.13	-4.4 (-9.8, 1.0)	-8.3 (-14.7, -1.9)*	0.29	-4.0 (-9.4, 1.5)	-5.3 (-11.8, 1.1)	0.19
	CG	2.3 (-3.1, 7.7)	Reference		3.9 (-1.6, 9.4)	Reference		1.4 (-4.2, 6.9)	Reference	
Endocrine symptoms	IG	-4.4 (-8.0, -0.9)*	-0.5 (-4.7, 3.8)	0.03	-5.5 (-9.1, -1.9)**	-0.3 (-4.6, 4.0)	0.02	-7.2 (-10.8, -3.5)**	-3.1 (-7.5, 1.3)	0.18
	CG	-4.0 (-7.6, -0.3)*	Reference		-5.2 (-8.9, -1.5)**	Reference		-4.1 (-7.8, -0.4)*	Reference	

* p < .05; ** p < .01; IG: Intervention group; CG: Control group; ES: Effect size

Supplementary Table S2: Effects of the PREFERABLE-EFFECT exercise program in subgroups of patients by age

		Change from Baseline to 3 months			Change from Baseline to 6 months			Change from Baseline to 9 months		
BR45 Scale		Within-group differences	Between-group differences	ES	Within-group differences	Between-group differences	ES	Within-group differences	Between-group differences	ES
**Age ≤ 50**										
Sexual function	IG	1.8 (-2.9, 6.4)	7.7 (1.2, 14.2)*	0.38	-0.4 (-5.1, 4.4)	7.9 (1.2, 14.5)*	0.39	-3.3 (-8.1, 1.5)	1.4 (-5.4, 8.3)	0.07
	CG	-5.9 (-11.2, -0.6)*	Reference		-8.2 (-13.7, -2.7)**	Reference		-4.7 (-10.3, 0.9)	Reference	
Sexual enjoyment	IG	9.7 (0.8, 18.6)*	14.5 (2.0, 27.0)*	0.57	9.5 (0.3, 18.8)*	10.1 (-3.8, 23.9)	0.40	9.7 (-0.3, 19.7)	15.6 (1.2, 29.9)*	0.61
	CG	-4.8 (-15.2, 5.6)	Reference		-0.5 (-12.3, 11.2)	Reference		-5.9 (-17.6, 5.8)	Reference	
Vaginal symptoms	IG	-0.8 (-7.4, 5.7)	-6.9 (-15.9, 2.2)	0.24	-9.6 (-16.2, -3.0)**	-12.6 (-21.9, -3.4)**	0.44	-5.7 (-12.5, 1.1)	-10.9 (-20.5, -1.4)*	0.38
	CG	6.0 (-1.5, 13.5)	Reference		3.0 (-4.7, 10.8)	Reference		5.3 (-2.7, 13.2)	Reference	
Skeletal symptoms	IG	2.1 (-4.8, 8.9)	-0.8 (-10.2, 8.6)	0.03	3.1 (-3.8, 9.9)	2.8 (-6.8, 12.3)	0.11	2.5 (-4.5, 9.5)	-1.0 (-10.8, 8.8)	0.04
	CG	2.9 (-4.9, 10.6)	Reference		0.3 (-7.7, 8.3)	Reference		3.5 (-4.6, 11.7)	Reference	
**Age > 50 to 60**										
Sexual function	IG	1.6 (-3.8, 7.1)	-0.4 (-7.3, 6.4)	0.02	2.2 (-3.5, 8.0)	4.6 (-2.5, 11.8)	0.23	3.5 (-2.2, 9.2)	7.7 (0.4, 15.0)*	0.39
	CG	2.0 (-3.0, 7.1)	Reference		-2.4 (-7.5, 2.7)	Reference		-4.2 (-9.6, 1.2)	Reference	
Sexual enjoyment	IG	1.4 (-8.1, 11.0)	-2.8 (-15.2, 9.5)	0.11	3.4 (-6.4, 13.2)	4.1 (-8.8, 16.9)	0.16	7.1 (-2.9, 17.2)	5.5 (-8.4, 19.3)	0.21
	CG	4.3 (-5.7, 14.3)	Reference		-0.6 (-11.2, 10.0)	Reference		1.7 (-9.9, 13.3)	Reference	
Vaginal symptoms	IG	-2.6 (-8.1, 2.9)	-1.7 (-8.6, 5.2)	0.06	-0.0 (-5.7, 5.7)	-0.8 (-7.9, 6.3)	0.03	-1.3 (-7.0, 4.5)	-0.4 (-7.6, 6.8)	0.01
	CG	-0.9 (-5.9, 4.2)	Reference		0.8 (-4.3, 5.9)	Reference		-0.9 (-6.2, 4.4)	Reference	
Skeletal symptoms	IG	-4.9 (-11.7, 1.9)	-1.5 (-9.9, 7.0)	0.06	-1.3 (-8.4, 5.7)	3.0 (-5.7, 11.7)	0.12	-2.7 (-9.7, 4.4)	-1.1 (-9.8, 7.7)	0.04
	CG	-3.5 (-9.7, 2.8)	Reference		-4.3 (-10.7, 2.0)	Reference		-1.6 (-8.0, 4.8)	Reference	
**Age > 60**										
Sexual function	IG	2.5 (-1.4, 6.3)	3.3 (-2.1, 8.7)	0.16	2.4 (-1.5, 6.3)	4.9 (-0.8, 10.7)	0.25	1.5 (-2.6, 5.6)	4.2 (-1.5, 9.9)	0.21
	CG	-0.8 (-4.9, 3.3)	Reference		-2.6 (-7.0, 1.9)	Reference		-2.7 (-6.9, 1.6)	Reference	
Sexual enjoyment	IG	12.4 (-1.0, 25.9)	4.7 (-14.8, 24.2)	0.18	6.8 (-4.8, 18.4)	0.8 (-17.6, 19.3)	0.03	13.5 (1.5, 25.4)	4.9 (-13.3, 23.2)	0.19
	CG	7.7 (-6.9, 22.4)	Reference		5.9 (-9.0, 20.9)	Reference		8.6 (-5.2, 22.4)	Reference	
Vaginal symptoms	IG	-3.0 (-8.5, 2.4)	-1.2 (-8.9, 6.5)	0.04	-4.7 (-10.2, 0.8)	-8.7 (-16.6, -0.7)*	0.30	-3.2 (-8.9, 2.5)	-3.5 (-11.4, 4.4)	0.12
	CG	-1.8 (-7.5, 3.9)	Reference		4.0 (-2.1, 10.1)	Reference		0.3 (-5.6, 6.1)	Reference	
Skeletal symptoms	IG	-6.6 (-12.9, -0.3)*	-9.9 (-18.7, -1.2)*	0.39	-6.8 (-13.1, -0.5)*	-12.3 (-21.3, -3.3)**	0.48	-5.3 (-11.8, 1.1)	-10.8 (-19.8, -1.9)*	0.42
	CG	3.3 (-3.3, 10.0)	Reference		5.5 (-1.5, 12.5)	Reference		5.5 (-1.3, 12.3)	Reference	

* p < .05; ** p < .01; IG: Intervention group; CG: Control group; ES: Effect size
